# A Cross-Sectional Study Investigating Lumbar Proprioception Impairments in Individuals with Type 2 Diabetes Mellitus: Correlations with Glycated Hemoglobin Levels

**DOI:** 10.3390/biomedicines11072068

**Published:** 2023-07-23

**Authors:** Mohammad A. ALMohiza, Ravi Shankar Reddy, Batool Abdulelah Alkhamis, Nabeel Hamdan Alghamdi, Adel Alshahrani, Bhaskar Reddy Ponneru, Debjani Mukherjee

**Affiliations:** 1 Department of Rehabilitation Sciences, College of Applied Medical Sciences, King Saud University, Riyadh 11433, Saudi Arabia; mmohiza@ksu.edu.sa; 2 Department of Medical Rehabilitation Sciences, College of Applied Medical Sciences, King Khalid University, Abha 61421, Saudi Arabia; balkamees@kku.edu.sa (B.A.A.); debjani@kku.edu.sa (D.M.); 3 Department of Physical Therapy, Faculty of Medical Rehabilitation Sciences, King Abdulaziz University, Jeddah 21589, Saudi Arabia; nhsalghamdi@kau.edu.sa; 4 Department of Medical Rehabilitation Sciences-Physiotherapy Program, College of Applied Medical Sciences, Najran University, Najran 55461, Saudi Arabia; amsalshahrani@nu.edu.sa; 5 Department of Physiotherapy, Janardan Rai Nagar Rajasthan Vidyapeeth University, Udaipur 313001, Rajasthan, India; bponneru@moh.gov.sa

**Keywords:** type 2 diabetes mellitus, lumbar proprioception, joint position sense, glycated hemoglobin, musculoskeletal health, falls prevention

## Abstract

Impaired proprioception is a recognized complication in individuals with type 2 diabetes mellitus (T2DM), contributing to balance deficits and increased risk of falls. However, limited research has focused on lumbar proprioception in this population. This study aimed to investigate lumbar proprioception in individuals with T2DM, as well as healthy individuals. Additionally, this study aimed to examine the correlation between lumbar proprioception and glycated hemoglobin (HbA1c) levels, which is a marker of long-term glycemic control in T2DM. A cross-sectional study was conducted, comparing lumbar joint reposition errors (JRE) between a T2DM group (*n* = 85) and a healthy group (*n* = 85). Lumbar JRE was assessed in flexion, extension, lateral bending left, and lateral bending right using a dual inclinometer device. HbA1c levels were measured as an indicator of glycemic control. Significant differences in lumbar JRE were found between the T2DM and healthy groups, with individuals with T2DM exhibiting larger JRE values, indicating impaired lumbar proprioception (*p* < 0.001). The correlation analysis revealed significant positive associations between HbA1c levels and lumbar JRE. Higher HbA1c levels were correlated with greater joint JRE in flexion (r = 0.49, *p* < 0.001), extension (r = 0.51, *p* < 0.001), left lateral bending (r = 0.45, *p* < 0.001), and right lateral bending (r = 0.48, *p* < 0.001) in the T2DM group. This study provides evidence of impaired lumbar proprioception in individuals with T2DM, as evidenced by larger lumbar JRE compared to the healthy group.

## 1. Introduction

Type 2 diabetes mellitus (T2DM) is a long-term metabolic condition marked by high levels of glucose in the blood [1]. It occurs as a result of the body’s reduced responsiveness to insulin and the decreased ability of the pancreas to produce sufficient insulin [1]. It is a global health concern, increasing worldwide prevalence [2]. As per data from the International Diabetes Federation, around 463 million adults were reported to have diabetes in 2019 [3]. It is projected that this number will increase to approximately 700 million by the year 2045 [3]. T2DM is linked with various complications, including musculoskeletal, cardiovascular disease, and neuropathy [4]. In recent years, researchers have begun to recognize the impact of T2DM on musculoskeletal health and functional outcomes [5].

Impaired proprioception, the sense of body position and movement, has been identified as a potential complication in individuals with T2DM [6,7]. Proprioception is crucial in motor control, postural stability, and movement coordination [7,8,9,10,11,12,13,14,15,16,17,18,19,20,21,22,23]. It relies on sensory information from specialized receptors in the muscles, tendons, and joints, which provide feedback to the central nervous system, enabling accurate perception and control of limb and body position [24]. Dysfunction of proprioceptive mechanisms can lead to balance deficits, altered movement patterns, and an increased risk of falls [24,25]. Though impaired proprioception has been extensively studied in the context of peripheral neuropathy in individuals with diabetes, particularly affecting the lower extremities [6,7,26], a paucity of research focuses specifically on lumbar proprioception in individuals with T2DM. The lumbar spine is critical in providing stability and transmitting forces between the upper and lower body during various activities [27]. Impaired lumbar proprioception may have significant implications for postural control, functional performance, and the development of musculoskeletal disorders, including low back pain [14,28,29].

Understanding the status of lumbar proprioception in individuals with T2DM is essential for several reasons. Firstly, it can contribute to our knowledge of the underlying mechanisms contributing to balance deficits and falls in this population. Falls are a major concern among individuals with diabetes, as they can lead to fractures, reduced mobility, and an overall decline in quality of life [30,31]. By elucidating the role of impaired lumbar proprioception in falls risk, interventions can be developed to target this specific impairment and potentially reduce the incidence of falls [32]. Secondly, impaired lumbar proprioception may have implications for managing chronic low back pain in individuals with T2DM. Low back pain is a prevalent musculoskeletal complaint, and its association with diabetes has been suggested in previous studies [33]. Dysfunction of the lumbar proprioceptive system may contribute to altered movement patterns, muscle imbalances, and increased stress on the spine, potentially exacerbating low back pain symptoms [34]. By identifying and addressing lumbar proprioceptive deficits, healthcare professionals can develop more targeted and effective interventions for managing low back pain in individuals with T2DM.

Furthermore, investigating the relationship between lumbar proprioception and glycemic control, as measured by glycated hemoglobin (HbA1c) levels, is of particular interest. HbA1c is a widely used marker of long-term glycemic control and reflects average blood glucose levels over two to three months [35]. Poor glycemic control has been associated with various complications of diabetes, including neuropathy and microvascular damage [36]. It is plausible that elevated HbA1c levels may contribute to impaired lumbar proprioception in individuals with T2DM, although the specific mechanisms underlying this relationship are not yet fully understood. This cross-sectional study aims to investigate lumbar proprioception between T2DM and healthy individuals and its correlations with HbA1c levels in T2DM individuals. We will compare lumbar joint position sense between a T2DM group and a healthy group, focusing on the relationship between lumbar proprioception and glycemic control. To assess lumbar joint position sense, we will utilize a dual inclinometer device, which provides objective and reliable measurements of joint reposition errors (JRE) in various movement directions. This study’s findings can contribute valuable insights into understanding lumbar proprioception impairments in individuals with T2DM. By establishing the presence and extent of lumbar proprioceptive deficits, healthcare professionals can tailor interventions specifically targeting this impairment. Proprioceptive training programs may be developed to improve sensory feedback, enhance postural control, and reduce the risk of falls in individuals with T2DM [37]. Additionally, identifying the correlations between lumbar proprioception and HbA1c levels can shed light on the influence of glycemic control on proprioceptive function. This knowledge may prompt the integration of strategies to optimize glycemic control as part of comprehensive management approaches for individuals with T2DM, potentially leading to improved musculoskeletal health and functional outcomes.

## 2. Materials and Methods

### 2.1. Study Design and Setting

From April 2021 to May 2023, a cross-sectional study was carried out at a tertiary healthcare center. The study adhered to the ethical principles set forth in the Declaration of Helsinki and obtained approval from the University Ethics Committee (approval number: Rec#234-987-435). In accordance with the CONSORT (Consolidated Standards of Reporting Trials) guidelines, this study followed the recommended reporting standards for clinical trials to ensure transparency and rigor in study reporting.

### 2.2. Participants

A total of 170 participants (85—T2DM and 85 asymptomatic) were recruited for the study through convenience sampling. The inclusion criteria were as follows: (1) diagnosed with T2DM according to the American Diabetes Association criteria [38], (2) aged between 40 to 80 years, and (3) able to provide informed consent. T2DM subjects were excluded if they had significant diabetic complications affecting balance or proprioception (e.g., peripheral neuropathy with foot ulcers), had any musculoskeletal or neurological conditions affecting the lumbar spine or lower extremities, or inability to understand or follow the study procedures. The inclusion criteria for the healthy control group in this study were as follows: (1) absence of a diagnosis of T2DM according to the American Diabetes Association criteria [38], (2) age between 40 and 80 years, and (3) able to provide informed consent. The healthy control group was excluded if they had any known musculoskeletal or neurological conditions affecting the lumbar spine or lower extremities, any significant medical conditions that could impact proprioception or balance, or demonstrated an inability to understand or follow the study procedures. The exclusion criteria aimed to ensure that the control group consisted of individuals without T2DM and without any underlying health conditions that could potentially affect lumbar proprioception or introduce confounding factors into the study. The participants for this study were recruited from multiple sources to ensure a diverse and representative sample. Recruitment efforts targeted both the T2DM group and the healthy group. For the T2DM group, individuals were identified and invited to participate in local diabetes clinics and community health centers in the neighborhood. Healthcare providers at these facilities were informed about the study and assisted in identifying eligible participants based on their medical records and diagnosis of T2DM. Potential participants were contacted either in person during their clinic visits or via phone to provide them with detailed information about the study and invite them to participate. To recruit participants for the healthy group, various strategies were employed. Online advertisements were placed on reputable health and wellness websites, social media platforms, and community forums, specifically targeting individuals without a diagnosis of T2DM or any known musculoskeletal symptoms. The advertisements provided a brief overview of the study and a link to a dedicated study website where interested individuals could find more information and express their interest in participation. All potential participants, both from the T2DM group and the healthy group, underwent a thorough screening process to determine their eligibility. The screening process included assessments of medical history, current health status, and confirmation of the inclusion and exclusion criteria. Eligible participants were then scheduled for a comprehensive assessment at the designated study site. Throughout the recruitment process, emphasis was placed on obtaining informed consent from each participant. Before collecting data, thorough information about the study, including its objectives, procedures, potential risks, and benefits, was provided to all participants. They were given ample opportunity to ask questions and clarify any concerns before providing their written informed consent to participate. Recruitment efforts were conducted over a specified time period, and potential participants were enrolled on a rolling basis as they met the eligibility criteria and expressed their willingness to participate. The demographic characteristics, apart from age, were not specifically monitored and matched between the diabetic and control groups. As a result, we cannot assert precise similarity between the two groups in terms of other demographic characteristics.

### 2.3. Lumbar Proprioception Assessment

Lumbar proprioception, which involves perceiving and controlling the position and movement of the lumbar spine, was assessed using a standardized protocol [39]. Participants followed established guidelines, starting in a comfortable standing posture with a target lumbar position as a reference. The primary researcher, trained in the assessment technique, ensured participants understood the task instructions and performed a practice trial.

**Figure 1:**
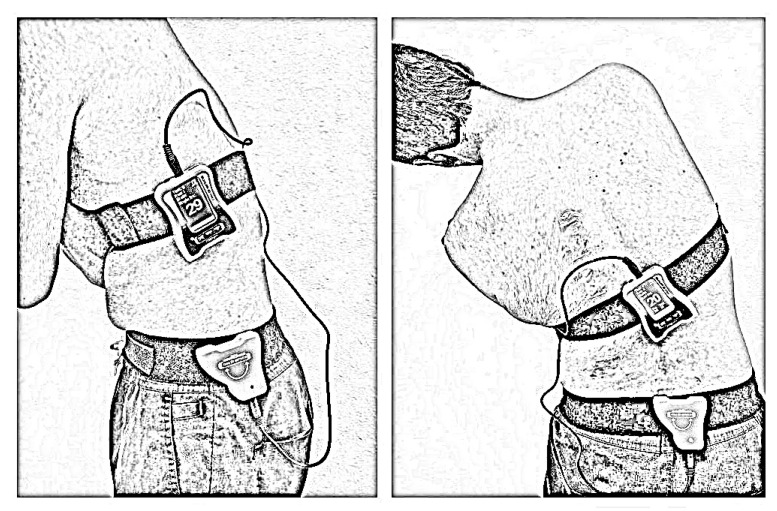
Assessment of lumbar joint reposition error in flexion, extension, left lateral bending, and right lateral bending.

In the study, lumbar JRE was measured in degrees for lumbar flexion, extension, and lateral bending in both directions to estimate lumbar JRE. The tests were conducted in a calm and well-ventilated laboratory, with subjects blindfolded during lumbar JRE testing. The Lumbar Proprioception Assessment, a well-established and reliable method for measuring lumbar spine movement and proprioceptive acuity, was utilized in this study. The reliability of this assessment has been extensively examined, with reported correlation coefficients ranging from 0.75 to 0.92 [39]. To measure lumbar JRE in flexion and extension, the primary inclinometer was placed on the chest at the T12 level, and the secondary inclinometer was positioned on the hemipelvis at the S1 level (Figure 1). 

For lateral bending left and right, the primary inclinometer was placed on the upper back (T12 spinous process) and the secondary inclinometer was placed on the sacrum. The subjects’ full range of motion was measured for flexion, extension, and lateral bending, and 50% of the available ROM was selected as the target position for the repositioning task. Participants were instructed to perform specific lumbar movements smoothly and comfortably while maintaining control, including flexion, extension, and lateral bending in both directions. The testing began with subjects standing straight and determining their self-selected neutral spine position. The examiner guided them to the target position (50% ROM) for five seconds, which the subjects memorized. They were then guided back to the starting position. Next, the subjects actively repositioned their lumbar spine to the target position and indicated completion by saying “YES.” Reposition errors were calculated as lumbar JRE in degrees. During each movement, the inclinometer recorded the angular changes in the lumbar spine, and the data in degrees were captured and stored for further analysis. The difference between the target angle and the participant’s reproduced angle was measured as proprioceptive accuracy. Each test was repeated thrice, and the mean value was used to measure repositioning accuracy.

### 2.4. Glycated Hemoglobin (HbA1c) Measurement

Blood samples were collected from the study participants after an 8-hour fast, ensuring that the samples were obtained under standardized conditions. The 8-hour fasting period helped to minimize potential variations in blood glucose levels due to recent meals. The blood samples were collected using venipuncture by a trained phlebotomist following standard aseptic techniques.

### 2.5. Sample Size Calculation

The sample size for this study was determined using G*Power software (version 3.1) based on the primary outcome measure, lumbar JRE. The calculation considered a two-sample t-test, with a power of 0.80 and an alpha level of 0.05. Due to limited previous studies investigating JRE in individuals with T2DM, estimating the effect size proved challenging [40]. A moderate effect size of 0.5 was considered for the calculation. Based on these parameters, it was determined that a minimum of 85 participants in both the T2DM and healthy groups would be necessary to detect a significant difference in lumbar JRE between the two groups.

### 2.6. Data Analysis

The normal distribution of the study variables was confirmed through the Shapiro–Wilk test, allowing for the application of parametric statistical analyses. Descriptive statistics were employed to summarize the demographic and clinical characteristics of the study participants, with continuous variables presented as means accompanied by standard deviations. To compare lumbar proprioception between individuals with T2DM and healthy individuals, an independent t-test was utilized. In addition, we calculated the effect size in terms of Cohen’s d. Cohen’s d is an estimate of effect size, which measures the standardized difference between two means after dividing the result by the pooled standard deviation. A Cohen’s d of 0.21 to 0.49 indicates a small effect size, 0.5 to 0.79 indicates a medium effect size and 0.8 or higher suggests a large effect size. Minimal detectable change (MDC) is computed to differentiate between random measurement error and real change. MDC was calculated as follows: (Standard Error Mean (SEM) × 1.65 × √2). Furthermore, the correlation between lumbar proprioception and HbA1c levels in T2DM was evaluated using correlation analysis, employing Pearson’s correlation coefficient. The strength of the correlation was interpreted according to established guidelines [41]. SPSS (version 24) was employed for data computation, and a significance level of *p* < 0.05 was applied to determine statistical significance.

## 3. Results

The results presented in Table 1 compare the demographic and clinical characteristics of the T2DM group (*n* = 85) and the healthy group (*n* = 85). 

The table includes variables such as age, gender, body mass index (BMI), duration of diabetes, and glycosylated hemoglobin (HbA1c) levels. The p-values indicate the statistical significance of any differences observed between the two groups. The T2DM group had an average HbA1c level of 7.46 ± 0.63. 

The results from Table 2 indicate significant differences in lumbar joint reposition error (JRE) between the T2DM group and the healthy group, which exhibited larger JRE values in flexion, extension, left lateral bending, and right lateral bending compared to the healthy group, indicating impaired lumbar proprioception in individuals with type 2 diabetes mellitus (T2DM). In flexion, the T2DM group had a significantly higher mean JRE (4.83 ± 1.23 degrees) compared to the healthy group (1.98 ± 0.99 degrees) (*p* < 0.001). Similar significant differences were observed in extension, left lateral bending, and right lateral bending, with the T2DM group showing larger JRE values compared to the healthy group (*p* < 0.001 for all comparisons). The effect sizes (Cohen’s d) for all comparisons were large, ranging from 1.36 to 1.93, suggesting substantial differences between the two groups. Additionally, the 95% confidence intervals of the difference did not overlap with zero, further supporting the statistically significant differences observed. The standard errors of the mean ranged from 0.13 to 0.19 degrees, indicating the precision of the estimated means. The minimal detectable change values ranged from 0.37 to 0.44 degrees, representing the smallest detectable difference that can be considered real change beyond measurement error.

**Figure 2:**
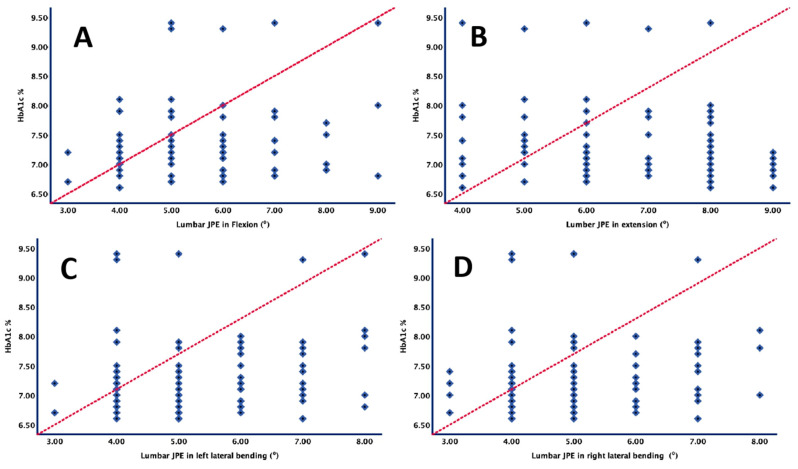
Correlation between HbA1c (%) values and lumbar joint reposition errors in (**A**) flexion, (**B**) extension, (**C**) left lateral bending and (**D**) right lateral bending.

Table 3 and Figure 2 present the coefficients of correlation between glycated hemoglobin (HbA1c) levels and lumbar JRE, specifically in flexion, extension, left lateral bending, and right lateral bending. 

The results of the correlation analysis demonstrated significant positive correlations between glycated hemoglobin (HbA1c) levels and lumbar joint reposition errors (JRE) in flexion, extension, left lateral bending, and right lateral bending. In flexion, higher HbA1c levels were associated with greater JRE (r = 0.49, *p* < 0.001). Similar correlations were observed in extension (r = 0.51, *p* < 0.001), left lateral bending (r = 0.45, *p* < 0.001), and right lateral bending (r = 0.48, *p* < 0.001). These findings indicate that elevated HbA1c levels, reflecting poorer glycemic control, are linked to compromised lumbar proprioception in individuals under investigation.

## 4. Discussion

The present study investigated the relationship between lumbar JRE, glycated hemoglobin (HbA1c) levels, and type 2 diabetes mellitus (T2DM). The results revealed several key findings that contribute to our understanding of lumbar proprioception in individuals with T2DM. The first important finding of this study is the impaired lumbar joint position sense observed in individuals with T2DM compared to the healthy group. The T2DM group exhibited significantly larger lumbar JRE in flexion, extension, left lateral bending, and right lateral bending, indicating compromised proprioceptive function. The findings revealed that individuals with T2DM exhibited significantly larger lumbar JRE in flexion, extension, left lateral bending, and right lateral bending compared to the healthy group. These results indicate compromised lumbar proprioception in individuals with T2DM, suggesting an impairment in their ability to accurately perceive and reproduce lumbar joint positions. 

The significant differences in lumbar JRE between the T2DM and healthy groups highlight the impact of diabetes on proprioceptive function. Impaired proprioception can have profound implications for postural control, movement coordination, and overall musculoskeletal health [42]. Accurate proprioceptive feedback is crucial for maintaining postural stability and preventing falls, particularly in dynamic and challenging situations [28,43]. The compromised lumbar proprioception observed in individuals with T2DM may contribute to an increased risk of falls and musculoskeletal injuries in this population. These findings align with previous research demonstrating proprioceptive deficits in individuals with diabetes in various joint regions, including the hip, knee, and ankle [7,44,45]. The compromised lumbar proprioception observed in this study may be attributed to the peripheral neuropathy commonly associated with T2DM, which affects sensory nerve fibers and impairs the transmission of proprioceptive signals [46]. The results of this study also provide insights into the specific joint positions where lumbar proprioceptive deficits are most prominent. The larger JRE values in flexion, extension, left lateral bending, and right lateral bending suggest a generalized impairment of lumbar proprioception in individuals with T2DM [25,43]. This implies that the ability to accurately perceive and control lumbar movements is compromised across multiple planes of motion [25]. 

The significant positive correlations between HbA1c levels and lumbar JRE further highlight the influence of glycemic control on proprioceptive function. Higher HbA1c levels, indicative of poorer long-term glycemic control, were associated with greater lumbar JRE in flexion, extension, left lateral bending, and right lateral bending. These findings suggest that chronic hyperglycemia and the associated microvascular and neural changes may contribute to the progressive impairment of lumbar proprioception in individuals with T2DM [47,48]. The observed correlations between HbA1c levels and lumbar JRE support previous studies that have reported similar associations in other joints [7,26,44]. Asiri et al. [7] discovered a significant positive correlation between HbA1c levels and ankle proprioceptive deficits in individuals with diabetes. In addition, the study revealed significant positive correlations between HbA1c values and hip JRE in multiple directions: hip flexion (r = 0.43, *p* < 0.001) and hip abduction (r = 0.36, *p* < 0.001). Similarly, in a study conducted by Reddy et al. [6], it was observed that participants with type 2 diabetes exhibited a significant moderate positive association between HbA1c levels and cervical joint repositioning errors (JREs) in flexion (r = 0.41, *p* = 0.001), extension (r = 0.48, *p* < 0.001), left rotation (r = 0.38, *p* < 0.001), and right rotation (r = 0.37, *p* < 0.001). This suggests that higher HbA1c levels in individuals with T2D are related to impaired cervical joint position sense, potentially impacting their cervical motor control and range of motion. Ettinger et al. [44] reported a positive correlation between HbA1c levels and knee JRE in individuals with T2DM. These consistent findings across different joint regions suggest impaired proprioception may be a generalized effect of chronic hyperglycemia and has broader implications for postural control and movement coordination in individuals with diabetes.

This study holds significant clinical implications for healthcare professionals involved in the management of individuals with T2DM. The findings highlight the presence of compromised lumbar proprioception in individuals with T2DM and emphasize the importance of addressing this issue in clinical practice. Firstly, the identification of impaired lumbar proprioception in individuals with T2DM suggests that healthcare professionals should prioritize the assessment of proprioceptive function in routine clinical evaluations. Including specific tests and measures to evaluate lumbar proprioception can provide valuable information about the patient’s motor control, postural stability, and risk of musculoskeletal complications. By incorporating proprioceptive assessments into regular clinical practice, healthcare professionals can identify individuals who may benefit from targeted interventions to improve proprioceptive function. Secondly, lumbar proprioception plays a crucial role in maintaining spinal stability, coordinating trunk movements, and protecting against low back pain [49]; impaired proprioception in these key lumbar movement patterns may contribute to chronic low back pain and functional limitations commonly observed in individuals with T2DM [50]. The findings from this study emphasize the need for interventions targeting proprioceptive deficits in individuals with T2DM. Proprioceptive training programs, focusing on enhancing sensory feedback and motor control, may help improve lumbar proprioception and mitigate the risk of falls and musculoskeletal complications [51,52]. Previous research has shown the potential benefits of proprioceptive training in improving balance, postural stability, and functional outcomes in individuals with diabetes [31,53]. Incorporating targeted exercises that challenge lumbar proprioception into diabetes management programs may contribute to improved motor control, reduced fall risk, and enhanced overall quality of life in individuals with T2DM [54].

Furthermore, addressing lumbar proprioceptive deficits in individuals with T2DM may have broader implications for the prevention of chronic low back pain. Low back pain is a common musculoskeletal complaint among individuals with diabetes, and compromised lumbar proprioception may contribute to its development and persistence [51]. By targeting lumbar proprioception as part of the management approach, healthcare professionals may be able to reduce the burden of chronic low back pain in individuals with T2DM and improve their overall quality of life. Moreover, the clinical significance of this study extends to fall prevention strategies in individuals with T2DM. Falls are a major concern among older adults with diabetes, and impaired proprioception is a known risk factor for falls [55]. By addressing lumbar proprioceptive impairments and improving postural control, healthcare professionals can play a vital role in reducing the risk of falls and fall-related injuries in individuals with T2DM [56,57]. This may involve incorporating proprioceptive training, balance exercises, and education on home safety into diabetes management programs.

Several limitations should be acknowledged when interpreting the results of this study. Firstly, the cross-sectional design employed in this study limits our ability to establish causal relationships between HbA1c levels, lumbar proprioception, and T2DM. Longitudinal studies with larger sample sizes are warranted to better understand the temporal associations and potential causative mechanisms. Secondly, the study sample consisted of individuals with diagnosed T2DM, which may introduce selection bias and limit the generalizability of the findings to the broader population of individuals with diabetes. It would be valuable to include individuals with different diabetes durations and varying levels of glycaemia control to provide a more comprehensive understanding of the relationship between HbA1c levels and lumbar proprioception. Additionally, the measurement of lumbar JRE using the dual inclinometer unit has inherent limitations. Although the inclinometer device is a reliable and commonly used instrument for assessing joint position sense [58,59], it relies on participants’ subjective perception and their ability to accurately reproduce their perceived lumbar positions. This subjective element may introduce variability and potential measurement errors. Furthermore, the study did not account for potential confounding factors that may influence lumbar proprioception, such as age, comorbidities, and physical activity levels. These factors may have an impact on proprioceptive function and could have influenced the study results. Future research should aim to control for these confounders to better understand the specific relationship between HbA1c levels and lumbar proprioception in individuals with T2DM. Lastly, the study focused on lumbar proprioception and its correlation with HbA1c levels, but it did not explore the functional implications of impaired proprioception in individuals with T2DM. Understanding how compromised lumbar proprioception affects postural control, movement coordination, and the risk of musculoskeletal complications would provide valuable insights for developing targeted interventions. Despite these limitations, the findings of this study contribute to the existing body of knowledge regarding lumbar proprioception in individuals with T2DM. Further research addressing the aforementioned limitations and investigating potential interventions to improve proprioceptive function in this population is warranted. Such research could have important clinical implications for optimizing postural stability, reducing the risk of falls, and enhancing the overall musculoskeletal health of individuals with T2DM.

In addition to investigating lumbar proprioception in individuals with T2DM, this study also aimed to examine lumbar proprioception in healthy individuals. Furthermore, the study sought to explore the correlation between lumbar proprioception and HbA1c levels, which serve as a marker for long-term glycemic control in T2DM. The primary objective was to establish the relationship between glycemic control and lumbar proprioception in individuals with T2DM, contributing to the understanding of this association. Future research should focus on investigating the long-term impact of impaired lumbar proprioception in individuals with T2DM to elucidate potential clinical implications. The potential role of drugs such as liraglutide in improving impaired lumbar proprioception should be considered [60]. Although direct studies on liraglutide’s impact on lumbar proprioception are lacking, its positive effects on body composition and metabolic parameters in overweight and obese individuals with type 2 diabetes mellitus suggest a potential positive influence on overall musculoskeletal function. Further research is needed to explore the specific benefits of liraglutide or similar therapies on lumbar proprioception in the context of obesity and type 2 diabetes mellitus.

## 5. Conclusions

In conclusion, this study provides evidence of compromised lumbar joint position sense in individuals with T2DM, indicating impaired lumbar proprioception compared to a healthy group. The significantly larger lumbar JRE observed in flexion, extension, left lateral bending, and right lateral bending in individuals with T2DM highlight the generalized nature of the proprioceptive deficits in this population. These findings have important clinical implications, as impaired lumbar proprioception can impact postural control, movement coordination, and overall musculoskeletal health. The results of this study contribute to the existing body of knowledge regarding proprioceptive impairments in individuals with T2DM, extending previous research that focused on other joint regions. The findings emphasize the need for targeted interventions aimed at improving lumbar proprioception in individuals with T2DM to reduce the risk of falls, musculoskeletal injuries, and chronic low back pain.

## Figures and Tables

**Table 1:** Characteristics of the diabetic and healthy population under investigation.

Variables	Diabetes Group (*n* = 85)	HealthyGroup (*n* = 85)	*p*-Value
Age in years	62.13 ± 4.82	61.42 ± 3.98	0.159
Gender ratio (*n*) M: F	46:39	45:40	0.186
Body mass index (kg/m^2^)	24.12 ± 3.26	23.33 ± 2.13	0.135
Duration of diabetes in years	7.63 ± 1.98	-	-
Glycosylated hemoglobin	7.46 ± 0.63	-	-

**Table 2:** Comparison of lumbar joint position between T2DM and healthy group.

Variables	T2DM Group (*n* = 85)	Healthy Group *(n* = 85)	95% CI of the Difference		Cohen’s d	SEM	MDC	*p*-Value
			Lower	Upper				
Lumber JRE in flexion (⁰)	4.83 ± 1.23	1.98 ± 0.99	0.96	3.01	1.36	0.13	0.39	<0.001
Lumbar JRE in extension (⁰)	5.33 ± 1.41	2.22 ± 1.13	1.21	3.92	1.74	0.16	0.44	<0.001
Lumbar JRE in lateral bending left (⁰)	4.56 ± 1.78	2.34 ± 1.11	1.23	3.01	1.66	0.16	0.39	<0.001
Lumbar JRE in lateral bending right (⁰)	4.23 ± 1.64	2.22 ± 1.08	1.76	2.94	1.93	0.19	0.37	<0.001

JRE = joint reposition error, T2DM = type 2 diabetes mellitus, CI = confidence interval, SEM = standard error of mean, MDC = minimal detectable change.

**Table 3:** Coefficient of correlation between HbA1c and lumbar joint reposition sense in T2DM individuals.

		Lumbar JRE in Flexion (⁰)	Lumbar JRE in Extension (⁰)	Lumbar JRE in Left Lateral Bending (⁰)	Lumbar JRE in Right Lateral Bending (⁰)
HbA1c %	r	0.49	0.51	0.45	0.48
	*p*	<0.001	<0.001	<0.001	<0.001

HbA1c = glycated hemoglobin, JRE = joint reposition error.

## Data Availability

The data will be provided on request and is available with the corresponding author (RSR).
